# Exploring the effects of supplementing monoterpenoids in *Moringa oleifera* based-diet in *Oreochromis niloticus:* Improving the growth performance, feed efficiency, digestibility and body composition

**DOI:** 10.1016/j.heliyon.2024.e34412

**Published:** 2024-07-09

**Authors:** Aqsa Sharif, Syed Makhdoom Hussain, Shafaqat Ali, Muhammad Rizwan, Khalid A. Al-Ghanim, Jean Wan Hong Yong

**Affiliations:** a Fish Nutrition Lab, Department of Zoology, Government College University Faisalabad, Faisalabad, Punjab, 38000, Pakistan; b Department of Environmental Sciences, Government College University Faisalabad, Faisalabad, Punjab, 38000, Pakistan; c Department of Biological Sciences and Technology, China Medical University, Taichung, 40402, Taiwan; d Department of Zoology, College of Science, King Saud University, Riyadh, 11451, Saudi Arabia; e Department of Biosystems and Technology, Swedish University of Agricultural Sciences, 23456, Alnarp, Sweden

**Keywords:** Moringa leaf meal, Monoterpenoids, Carvacrol, *Oreochromis niloticus*, Menthol, Health status

## Abstract

Monoterpenoids are interesting hydrocarbons typically found in essential oils and have a significant role in medicinal and biological purposes. The goal of this study was to investigate the effects of two monoterpenoids, carvacrol (CAR) and menthol (MEN), supplemented with *Moringa oleifera* leaf meal (MOLM) based diets on growth parameters, digestibility and body composition of Nile tilapia (*Oreochromis niloticus*). Alongside the basal diet (control-T1), nine experimental diets supplemented with categorized levels of CAR and MEN at 200, 300 and 400 mg/kg individually and their mixtures (MIX) (1:1) (CAR-T2, 200; T3, 300; T4, 400 mg/kg, MEN-T5, 200; T6, 300; T7, 400 mg/kg and MIX- (1:1) T8, 200; T9, 300; T10, 400 mg/kg) were fed to the fingerlings (6.55 ± 0.03 g) for the period of 60 days. Monoterpenoids supplementation led to significantly (*p*<0.05) better growth, feed utilization and nutrient digestibility in comparison to the control group. The highest growth, feed efficiency and nutrient digestibility were noticed in fishes fed with a diet supplemented with 200 mg/kg MIX. Interestingly, fishes fed with diets containing monoterpenoids had significantly higher levels of protein and ash, but with lower lipid in comparison to the control group. Conclusively, the dietary supplements like CAR and MEN improved the health status of Nile tilapia when given either individually or in a mixture,. Specifically, the MIX at 200 mg/kg was the optimal supplementation for the fishes.

## Graphical abstract

**Figure:**
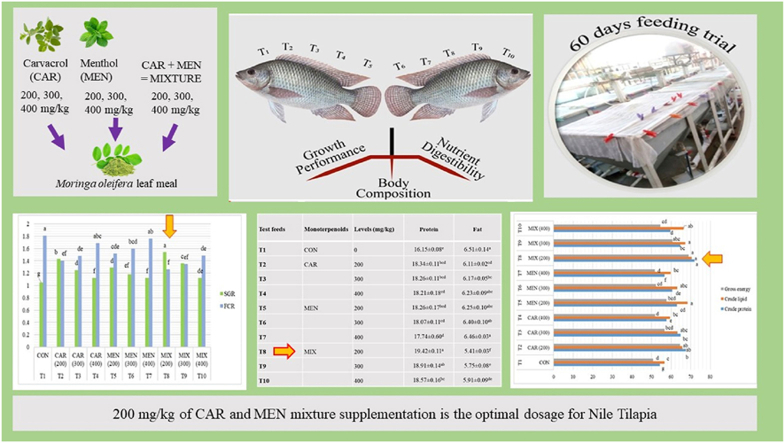

## 1 Introduction

As a component of the food production industry that provides humans with high-quality protein and fat, aquaculture is crucial to human health and wellbeing [1]. Important breakthrough in this industry occurred in 1970–1980s and developed increasingly across the globe [2]. Recently, field of fish nutrition is gaining great attention because species-specific artificial diets are being formulated to satisfy the nutritional demands of farmed fishes [3]. Nutrition is the most crucial domain in semi-intensive and intensive fish culture since feed accounts for 40–60 % of production costs and is thought to be the single largest expense in aquacultural production [4]. On commercial feed formulations, the protein need is usually satisfied by adding fishmeal (FM) which has the largest portion of protein content, beneficial lipids and other necessary nutrients. Unfortunately, there is scarcity of inland and oceanic resources to produce fish oil, FM and other protein sources for fish feed [[5], [6]]. Plant derived products are advantageous than FM as they are economical, contain a smaller number of pollutants and can be processed easily [7]. El-Saidy and Gaber [8] replaced FM in Nile tilapia (*Oreochromis niloticus*) with a blend of plant proteins. They found that in 16-week-old fingerlings, FM replacement was possible in 100 % of cases with no adverse effects.

Using natural supplements in aquaculture production is seen to be an effective measure to strengthen fish immunity and also creating a healthy environment [[9], [10]]. Various botanicals have a storehouse of useful biologicals; can serve as effective supplements to improve growth and immunity due to the abundance of bioactive components, including flavonoids, polysaccharides, saponins, polyphenols, essential oils, terpenoids and alkaloids [[10], [11], [12], [13], [14], [15]]. These natural substances have been utilized extensively in improving growth, organ and tissue function, nutrient metabolism, controlling bacterial and viral infections [[5], [7], [9], [16], [17]]. A member of Moringaceae family, moringa (*Moringa oleifera*), popularly known as the miracle tree or drumstick plant, is a resilient plant that can easily adapt to a number of climatic conditions and varied soil types [18]. Fruits, leaves, stem and roots of this plant are of high nutritional value and have anti-cancer, antioxidant, antibacterial, antifungal and anti-inflammatory properties [16]. Above all, leaves of moringa are rich source of carotenoids, vitamins, glucosinolates, flavonoids, alkaloids, isothiocyanates, saponins, phenolic acids, and tannins [[10], [15], [17], [19]]. A number of studies concerning fish growth and welfare have been successfully conducted by using leaves of this plant [20,[9], [21]]. Different researchers also conducted studies to check potential of *M. oleifera* leaf meal (MOLM) in *Labeo rohita* and *O. niloticus* and unveil that MOLM can be successfully used to substitute FM [22,23].

Usage of feed additives in the field of aquaculture is gaining enormous importance [[9], [24], [25]]. Phytogens are the dietary additives obtained from herbs, plants or spices and widely known for their role as natural growth promoters or non-antibiotic health regulators [26]. Monoterpenoids are secondary metabolites derived from essential oils. They have extensive medicinal properties i.e., antioxidant, antibacterial and anti-inflammatory [27]. The essential oil obtained from *Origanum vulgare*, a widely distributed aromatic plant, contains a significant amount of carvacrol (CAR), a monoterpenoid phenol [28]. It acts as an anti-microbial, health modulator, digestion booster and antioxidant enzyme activator agent [29]. Various researchers have found positive results of different phytogenic compounds such as CAR and thymol [30], cineole [24] and curcumin [31] on antioxidant defense system in fish under normal conditions. Menthol (MEN) is referred as major active ingredient present in essential oil of *Mentha piperita* [25]. In mammals, it acts as an antioxidant, anti-apoptotic and anti-inflammatory component [32]. Recently, in Nile tilapia MEN essential oil was reported to have a significant role to reduce toxicity caused by water pollutants like chlorpyrifos and activates anti-oxidative, immune and anti-inflammatory responses of fish. Growth performance also has been also improved by dietary MEN oil in Nile tilapia [33,34]. The Cichlidae is the family of Nile tilapia, *O. niloticus*, which is widely reared in freshwater around the world. Its husbandary practices range from common household ponds to high-end commercial products [35]. The biological features which make this fish feasible for farming are rapid reproductive rate, excellent fillet quality, acceptance of a variety of commercial diets, disease resistance [36] and tolerance to poor water quality. Therefore, utilizing *M. oleifera* leaf meal (MOLM) based diets, this research explored the effects of dietary CAR and MEN, individually and in combination, as well as the different doses, on the growth, body composition, and nutrient digestibility of Nile tilapia.

## 2 Materials and methods

### 2.1 Experimental diets

The diet components were acquired from a commercial mill in Faisalabad, Pakistan, and their ground contents were subjected to conventional techniques of the AOAC to determine their chemical composition for the preparation and formulation of test diets [37] (Table 1, Table 2). Monoterpenoids, CAR and MEN, were obtained from the Natural Products and Synthetic Chemistry Laboratory, Department of Applied Chemistry and Biochemistry, Government College University, Faisalabad, Pakistan. A basal diet (T1, control or without CAR and MEN) was formulated following the nutritional standards set by Ref. [38] and supplemented with graded levels of CAR (T2, 200; T3, 300; T4, 400 mg/kg), MEN (T5, 200; T6, 300; T7, 400 mg/kg) and MIX (1:1) (T8, 200; T9, 300; T10, 400 mg/kg) (Table 1) [39,40]. Sieve size of 0.5 mm diameter was used to prepare feed pellets particles. Prior to adding the fish oil, all components were well combined. The hydration level of the feed was kept-up to about 10–15 % and a proper textured dough was formed to prepare the floating pellets of 3 mm by a special laboratory extruder (Model SYSLG30-IV Experimental Extruder) [41]. CAR and MEN were added in the diets after mixing in fish oil, as described by Hoseini et al. [40]. The freshly prepared floating pellets were dried in oven and kept at 4 °C until further use.

**Table 1:** Formulation (%) of the basal diet.

Ingredients	T_1 (Control)_	T2	T3	T4	T5	T6	T7	T8	T9	T10
Carvacrol (CAR) (mg/kg)	0	200	300	400	–	–	–	–	–	–
Menthol (MEN) (mg/kg)	0	–	–	–	200	300	400	–	–	–
Mixture (MIX/CAR + MEN) (mg/kg)	0	–	–	–	–	–	–	200	300	400
*Moringa oleifera* leaf meal	52	52	52	52	52	5	52	52	52	52
Fish Meal	13	13	13	13	13	13	13	13	13	13
Wheat Flour	12	12	12	12	12	12	12	12	12	12
Fish Oil	7	7	7	7	7	7	7	7	7	7
Corn Gluten (60 %)	12	12	12	12	12	12	12	12	12	12
Vitamin Premix^a^	1	1	1	1	1	1	1	1	1	1
Ascorbic Acid	1	1	1	1	1	1	1	1	1	1
Chromic Oxide	1	1	1	1	1	1	1	1	1	1
Mineral premix^b^	1	1	1	1	1	1	1	1	1	1

a Vitamin (Vit.) premix kg^−1^: Vit. A: 15, 000,000 IU, Vit. C: 15,000 mg, Vit. B_2_: 7000 mg, Vit. E: 30000 IU, Ca pantothenate: 12,000 mg, Vit. B_12_: 40 mg, Vit. D_3_: 3,000,000 IU, Vit. B_6_: 4000 mg, Folic acid: 1500 mg, Vit. K_3_: 8000 mg, and Nicotinic acid: 60,000 mg.

b Mineral premix kg^−1^: Mg: 55 g, Ca: 155 g, Mn: 2000 mg, P: 135 g, I: 40 mg, Co: 40 mg, Na: 45 g, Se: 3 mg, Fe: 1000 mg, Cu: 600 mg, and Zn:3000 mg.

**Table 2:** Percentage chemical analysis of feed components (on dry matter basis).

Ingredients	Dry matter (%)	Gross Energy (kcal/g)	Crude Protein (%)	Ash (%)	Crude Fiber (%)	Crude Fat (%)	Carbohydrates (%)
Fish meal	91.53	4.07	46.17	24.23	1.13	6.15	18.25
Wheat flour	92.53	2.86	10.54	2.81	2.59	2.36	78.84
Corn gluten (60 %)	2.06	4.57	8.79	1.65	1.37	4.28	33.91
*Moringa oleifera* leaf meal	2.84	2.39	7.69	12.34	7.89	6.54	45.54

### 2.2 Fish rearing and maintenance

From the Fish Seed Hatchery in Faisalabad, Pakistan, a total of 450 *O. niloticus* fingerlings (6.55 ± 0.03 g) were procured. Two-week period was provided to fish for acclimatization to laboratory conditions and water quality parameters. A total of 30 V-shape tanks of 70-L volume were especially designed to collect fecal matter. Fifteen fish per tank were stocked in triplicates in specially designed Government College University Faisalabad aquaculture system. Before the experiment, to clean the fish from ectoparasites or fungus, they were immersed in NaCl solution of 5 g/L volume for 1–2 min [42]. The fish were acclimatized for a two-week period, during which they were fed the basal diet ad libitum once a day [43].

In a standing water system with continuous aeration, fingerlings were hand fed twice daily (08:00 a.m. and 2:00 p.m.) until they appeared satiated for 60 days during the feeding trial. After 20 min of feed application, remaining un-consumed diet was collected by opening valves of tanks so to determine the feed intake which will be used to assess the feed conversion ratio (FCR). Then the three fourth of tanks were emptied and fresh water was refilled. Preceding the 2 h of feed offering to the fish, fecal strings were gathered by fecal collecting tube from every tank and handed carefully to avoid mineral discharge. During the feeding trial, water quality parameters were regularly monitored and measured to ensure optimal conditions and water quality, e.g., dissolved oxygen (5.9–7.2 mg/L) by DO meter (Jenway 970), water temperature (24.9 ± 0.03–28.7 ± 0.01 °C) by thermometer and pH (7.4 ± 0.04–8.6 ± 0.02) by pH meter (Jenway 3510).

### 2.3 Growth assessment

The growth trial lasted for 60 days. The standard formulae for FCR, weight gain and specific growth rate (SGR) were utilized to calculate growth performance as determined by Ref. [20].

### 2.4 Chemical analysis

For analysis, three fish were sampled from each replicate by giving anaesthesia (clove oil) as described by Coyle et al. [44] and oven dried at 70 °C to estimate the weight loss. Chemical analysis of fish whole-body, diet and feces were carried out according to AOAC [37]. After homogenizing the fish and diets in a mortar and pestle, proximate analysis was carried out on them at the conclusion of the feeding experiment. The moisture content in the sample was assessed by drying them in oven at temperature of 105 °C for a time of 12 h. Using a micro Kjeldahl device, the crude protein (N × 6.25) was calculated. The bomb calorimeter data was utilized to calculate gross energy content (Parr Instrument Co., Moline, USA). The lipid content was determined using the petroleum ether extraction method [45] through Soxtec HT2 1045 system (40–60 °C); whereas crude ash was determined by igniting the samples at 650 °C for 12 h in an electric furnace (Eyela-TMF 3100) to constant weight.

### 2.5 Digestibility

The digestibility trial was conducted for 30 days for fecal collection. Using dietary chromic oxide as an inert marker at 1 % inclusion level, the digestibility coefficients (ADC) of nutrients like crude fat (CF), crude protein (CP) and gross energy (GE) were evaluated. The assessment of chromic oxide in feed and feces ash samples was done by oxidation with molybdate reagent using spectrophotometer (UV-VIS 2001) set to 370 nm absorbance, in accordance with the acid digestion method [46]. The ADC of experimental diets was evaluated by the equation proposed by National Research Council (NRC) [38].

### 2.6 Data analysis

The mean ± SD is used to express data. Levene's test was used to verify the homogeneity and normality of data. Prior to analysis, percentage data underwent an arcsine transformation. The effects of inclusion level (200, 300, and 400 mg/kg) and monoterpenoid type (CAR, MEN, and MIX) on target parameters were assessed using two-way analysis of variance (ANOVA). To determine significant differences between the treatments, all 10 groups were analyzed using a one-way ANOVA, and then Tukey's significant test was performed. A significant threshold of *p*<0.05 was set.

## 3 Results

### 3.1 Growth performance

**Fig. 1:**
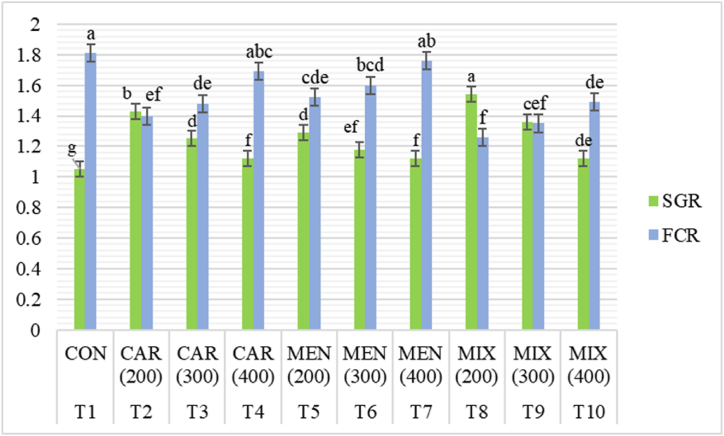
Effect of dietary supplementation of monoterpenoids on the SGR% and FCR of *O. niloticus*. CON = control, CAR = carvacrol, MEN = menthol, MIX = mixture.

The monoterpenoid type, inclusion level, and their interaction all had a notable (*p*<0.05) impact on the growth performance of *O. niloticus* (Table 3 and Fig. 1). The highest final weight (26.16 g), % weight gain (299.56 %) and SGR (1.54), and the lowest FCR (1.26) was noticed in T8 supplemented diet (1:1 MIX at 200 mg/kg) while the control diet (T2) had lowest final weight, % weight gain and SGR (16.94 g, 158.15 % and 1.05, respectively), and the highest FCR (1.81). With increasing levels of CAR, MEN, and MIX supplementation, growth performance declined and feed utilization tended to decline, with the highest value at 200 mg/kg concentration at each condition. But when compared to the control group, supplementing with monoterpenoids often produced greater growth and feed efficiency at any concentration. Throughout the feeding trial, there was no mortality.

**Table 3:** Effects of dietary carvacrol and menthol supplemented MOLM based diets on growth performance of *O. niloticus*. The data are presented as mean ± standard deviation. There is a considerable difference between data with different letters in the same column.

Test feeds	Monoterpenoids	Levels (mg/kg)	IW (g)	FW (g)	SGR	WG (g)	FCR
T1	CON	0	6.56 ± 0.03	16.94 ± 0.06^g^	1.05 ± 0.00^g^	158.15 ± 0.34^g^	1.81 ± 0.04^a^
T2	CAR	200	6.53 ± 0.02	23.62 ± 0.63^b^	1.43 ± 0.03^b^	261.33 ± 8.62^b^	1.40 ± 0.06^ef^
T3		300	6.55 ± 0.02	20.22 ± 0.30^de^	1.25 ± 0.02^d^	208.44 ± 4.31^de^	1.48 ± 0.03^de^
T4		400	6.53 ± 0.02	17.96 ± 0.11^g^	1.12 ± 0.00^f^	174.85 ± 0.56^fg^	1.69 ± 0.03^abc^
T5	MEN	200	6.55 ± 0.04	20.84 ± 0.83^d^	1.29 ± 0.05^d^	218.15 ± 0.76^d^	1.52 ± 0.04^cde^
T6		300	6.57 ± 0.02	19.18 ± 0.22^ef^	1.18 ± 0.02^ef^	190.23 ± 4.17^ef^	1.60 ± 0.13^bcd^
T7		400	6.58 ± 0.02	18.07 ± 0.07^fg^	1.12 ± 0.01^f^	174.35 ± 1.89^fg^	1.76 ± 0.04^ab^
T8	MIX	200	6.54 ± 0.03	26.16 ± 0.55^a^	1.54 ± 0.03^a^	299.56 ± 9.15^a^	1.26 ± 0.04^f^
T9		300	6.55 ± 0.03	22.20 ± 0.37^c^	1.36 ± 0.02^c^	239.00 ± 6.75^c^	1.35 ± 0.08^ef^
T10		400	6.55 ± 0.01	19.72 ± 0.14^de^	1.12 ± 0.01^de^	200.97 ± 1.97^de^	1.49 ± 0.10^de^
*2-way ANOVA*							
Monoterpenoid			0.0404*	0.0000***	0.000***	0.000***	0.000***
Level			0.2459ns	0.0000***	0.000***	0.000***	0.000***
Monoterpenoid × Level			0.4347ns	0.0000***	0.000***	0.844ns	0.000***

CON = Control, CAR = Carvacrol, MEN = Menthol, MIX = Mixture (carvacrol + menthol) (1:1). Data are means of triplicate.

### 3.2 Nutrient digestibility

**Fig. 2:**
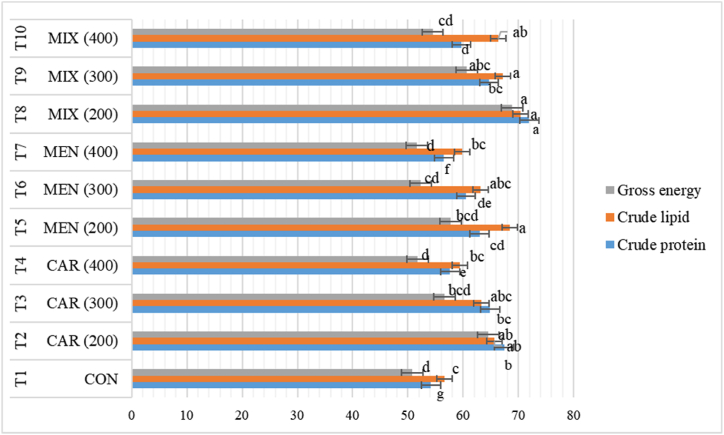
Effect of dietary monoterpenoids supplementation on the digestibility coefficient (%) of *O. niloticus*. CON = control, CAR = carvacrol, MEN = menthol, MIX = mixture.

Protein digestibility was significantly influenced by the monoterpenoid type, the inclusion level and their interaction, while lipid and energy digestibilities were affected significantly by the type of monoterpenoid and inclusion level (*p*<0.05) (Table 4, Table 5 and Fig. 2). The highest ADC for CP (71.96 %), CF (70.44 %) and GE (68.86 %) was noticed in fish fed T8 diet complemented with MIX (1:1) at 200 mg/kg whereas the control diet had the minimum ADC values for CP (54.18 %), CF (56.62 %) and GE (50.83 %). In comparison to control group, nutrient digestibility was significantly higher in monoterpenoids supplemented groups regardless of the type and level of supplementation, except for protein and energy digestibility in the group supplemented with MEN at the highest level; however, ADC decreased with increasing level of dietary monoterpenoids at all circumstances.

**Table 4:** Effects of dietary carvacrol and menthol supplemented MOLM based diets on analyzed diet content (%) of *O. niloticus*. Mean ± S.D. is provided for data. Significant differences exist between the data in the same column with different letters.

Test feeds	Monoterpenoids	Levels (mg/kg)	Crude protein	Crude lipid	Gross energy
T1	–	0	29.31 ± 0.03	7.42 ± 0.11	2.86 ± 0.04
T2	CAR	200	29.38 ± 0.02	7.36 ± 0.10	2.82 ± 0.04
T3		300	29.27 ± 0.02	7.37 ± 0.09	2.84 ± 0.04
T4		400	29.34 ± 0.04	7.28 ± 0.08	2.78 ± 0.04
T5	MEN	200	29.38 ± 0.04	7.31 ± 0.04	2.83 ± 0.06
T6		300	29.41 ± 0.03	7.32 ± 0.03	2.81 ± 0.06
T7		400	29.45 ± 0.06	7.35 ± 0.04	2.76 ± 0.06
T8	MIX	200	29.27 ± 0.05	7.28 ± 0.03	2.82 ± 0.04
T9		300	29.27 ± 0.04	7.39 ± 0.08	2.79 ± 0.04
T10		400	29.27 ± 0.06	7.37 ± 0.08	2.78 ± 0.07
*2-way ANOVA*					
Monoterpenoid			0.0000***	0.9148ns	0.8143ns
Level			0.0140*	0.8031ns	0.0215*
Monoterpenoid × Level			0.0231*	0.1438ns	0.6567ns

CON = Control, CAR = Carvacrol, MEN = Menthol, MIX = Mixture (carvacrol + menthol) (1:1). Data are the means of triplicate.

**Table 5:** Effects of dietary supplementation of carvacrol and menthol supplemented MOLM based diets on feces' content (%) of *O. niloticus*. Mean ± S.D. is provided for data. Significant differences exist between the data in the same column with different letters.

Test feeds	Monoterpenoids	Levels (mg/kg)	Crude protein	Gross energy	Crude lipid
T1	–	0	18.39 ± 0.10^a^	2.11 ± 0.06^a^	4.98 ± 0.12^a^
T2	CAR	200	9.69 ± 0.26^bc^	1.19 ± 0.07^bb^	2.17 ± 0.06^bb^
T3		300	12.44 ± 0.09^bd^	1.47 ± 0.09^bb^	2.78 ± 0.06^bd^
T4		400	15.22 ± 0.01^bb^	1.65 ± 0.08^ba^	3.11 ± 0.07^bc^
T5	MEN	200	10.69 ± 0.24^ac^	1.41 ± 0.04^ab^	2.45 ± 0.07^ab^
T6		300	13.90 ± 0.06^ad^	1.68 ± 0.06^ab^	2.88 ± 0.08^ad^
T7		400	16.90 ± 0.06^ab^	1.93 ± 0.04^aa^	3.38 ± 0.04^ac^
T8	MIX	200	8.90 ± 0.06^cc^	1.08 ± 0.08^cb^	1.87 ± 0.06^cb^
T9		300	10.90 ± 0.06^cd^	1.32 ± 0.05^cb^	2.03 ± 0.04^cd^
T10		400	12.90 ± 0.06^cb^	1.57 ± 0.05^ca^	2.45 ± 0.06^cc^
*2-way ANOVA*					
Monoterpenoid			0.0000***	0.0000***	0.0000***
Level			0.0000***	0.0000***	0.0000***
Monoterpenoid × Level			0.0000***	0.0000***	0.0000***

CON = Control, CAR = Carvacrol, MEN = Menthol, MIX = Mixture (carvacrol + menthol) (1:1). Data are means of triplicate.

### 3.3 Body composition

The type of monoterpenoid, inclusion level and their interactions significantly affected protein content, while lipid level was substantially (*p*<0.05) impacted by the type of monoterpenoid and inclusion level (Table 6). When compared to the control group, the addition of monoterpenoids led to a noticeably higher crude protein level at all concentrations. The T8 supplemented diet with 200 mg/kg of MIX (1:1) had the highest crude protein content (19.42 %). Similar with growth performance, crude protein diminished from body with increasing level of dietary CAR, MEN and MIX. The T8 diet had the lowest crude lipid (5.41 %), whereas fish fed the diet without supplementation (control) had maximum crude lipid (6.51 %).

**Table 6:** Effects of dietary carvacrol and menthol supplemented MOLM based diets on wet body composition (%) of *O. niloticus*. Mean ± S.D. is provided for data. Letter-differentiated data differ greatly from one another. Data are mean of triplicate.

Test feeds	Monoterpenoids	Levels (mg/kg)	Ash	Moisture	Protein	Fat
T1	CON	0	3.18 ± 0.08	73.15 ± 0.14	16.15 ± 0.08^e^	6.51 ± 0.14^a^
T2	CAR	200	3.39 ± 0.11	71.08 ± 0.05	18.34 ± 0.11^bcd^	6.11 ± 0.02^cd^
T3		300	3.40 ± 0.13	71.01 ± 0.07	18.26 ± 0.11^bcd^	6.17 ± 0.05^bc^
T4		400	3.37 ± 0.34	71.00 ± 0.05	18.21 ± 0.18^cd^	6.23 ± 0.09^abc^
T5	MEN	200	3.31 ± 0.13	71.03 ± 0.06	18.26 ± 0.17^bcd^	6.25 ± 0.10^abc^
T6		300	3.29 ± 0.09	71.02 ± 0.03	18.07 ± 0.11^cd^	6.40 ± 0.10^ab^
T7		400	3.25 ± 0.07	71.01 ± 0.54	17.74 ± 0.60^d^	6.46 ± 0.03^a^
T8	MIX	200	3.13 ± 0.08	71.09 ± 0.03	19.42 ± 0.11^a^	5.41 ± 0.03^f^
T9		300	3.25 ± 0.12	71.10 ± 0.05	18.91 ± 0.14^ab^	5.75 ± 0.08^e^
T10		400	3.35 ± 0.16	71.02 ± 0.05	18.57 ± 0.16^bc^	5.91 ± 0.09^de^
*2-way ANOVA*						
Monoterpenoid			0.062ns	0.061ns	0.000***	0.000***
Level			0.306ns	0.136ns	0.001***	0.000***
Monoterpenoid × Level			0.706ns	0.528ns	0.159ns	0.002***

CON = Control, CAR = Carvacrol, MEN = Menthol, MIX = Mixture (carvacrol + menthol) (1:1). Data are means of triplicate.

Crude lipid content tended to rise in contrast to crude protein content when dietary CAR, MEN, and MIX levels increased; the highest values were observed at 400 mg/kg concentration. The dietary interventions had no discernible effect on the amount of moisture or ash.

## 4 Discussion

Natural phytochemicals are receiving increasing importance in the field of aquaculture for their growth promoting, antioxidant and antibacterial potential [[9], [47]]. For example, dietary phytochemicals in rainbow trout, such as thymol and CAR [30] and mixture of CAR, thymol, anethol and limonene in channel catfish [48] have been reported as growth promoters and immune modulators.

In the current research, two monoterpenoid phytochemicals such as CAR and MEN were tested solely or in mixture at grading levels in Nile tilapia diets. Fish given a CAR and MEN supplemented diet at a 200 mg/kg level as MIX (1:1) showed the best growth, feed efficiency, and digestibility. However, although growth, feed utilization, and digestibility were always greater in fish given monoterpenoids supplemented diets than in the group without supplementation, they declined with increasing dietary monoterpenoids. Comparable to our research, Adel et al. [49] observed a dose-dependent rise in growth of Caspian white fish fed diets supplemented with MEN, indicating that 3 % of the food is the ideal amount. Similarly, Zheng et al. [50] found growth promoting effect of combination of thymol (0.0015 %) and CAR (0.0485 %) in channel catfish suggesting that MIX of phytochemicals is more effective compared to their individual use. Growth promoting effect of CAR was also reported in rainbow trout, with the ideal dietary level of 1.5 % being suggested [51]. According to Dawood et al. [52], MEN fortified feed resulted in improved growth parameters in Nile tilapia. The addition of MEN and CAR to fish feed may lead to increased digestive enzyme activity, which in turn suggests that the enhanced growth performance is largely due to improved feed intake and digestion, attributed to the effectiveness of menthol in stimulating appetite and nutrient absorption [30,53]. The basic mechanism behind growth improvement by herbal additives lies in the fact that decreased stress level in body leads to the improved energy budget and in turn assimilation and absorption of nutrients is stimulated [25]. Concurrent to our study, Dawood et al. [33] reported growth-promoting activity of MEN under chlorpyrifos toxicity at 0.2–0.3 % dietary levels in Nile tilapia. MEN was also reported to stimulate absorption and digestion activity in fish gut, improving feeding efficiency [34]. Additionally, MEN containing essential oil has a special fragrance which lures the fishes and makes feed more attractive [54].

Conversely, rainbow trout (*Oncorhynchus mykiss*) fed MEN enriched diets for 30 days showed a negligible growth response [40]. In the same way, dietary CAR had no discernible impact on rainbow trout growth or feed efficiency [30]. These contradicting results could be the consequence of differences in fish size, species, feeding period, and experimental circumstances.

In current study, significant variation in nutrient digestibility were observed in MEN and CAR supplemented diets. A possible reason behind the enhanced nutrient digestibility is the copious release of digestive enzymes [55], as dietary phytochemicals complemented at 5000 and 10,000 ppm enhanced production of protease, lipase and α-amylase in *O. mossambicus* [56]. Wenk [57] stated the function of phytochemicals as digestibility enhancer particles, which work by balancing the intestinal microbiota count and secreting the endogenous enzymes. Hence, positive outcomes in terms of nutrient digestibility and absorption are perceived, increasing the bioavailability of nutrients to the fish [30].

The dietary therapies in the current study had a considerable impact on body proximate composition. The MIX (1:1) supplemented diet resulted in fish with the highest protein content and the lowest fat content. When comparing a control diet to those supplemented with monoterpenoids, the latter showed a much higher level of protein. Similarly, Mansour et al. [58] reported increased protein content while the fat content was decreased with increasing level of dietary MEN in Nile tilapia. Peterson et al. [48] found low fat contents in channel catfish when employing phytogenic feed additive, which resulted in improved protein contents. Yilmaz et al. [59] proved that the thymol present in herbs such as fenugreek and rosemary lead to improved weight gain, growth rate, FCR and muscle protein of sea bass due to the release of pancreatic enzymes and activation of accessory factors needed for feed utilization. Opposing to our results, Hoseini et al. [40] described that dietary MEN did not influence body composition in rainbow trout. The role of genistein and resveratrol (phytochemicals) on the digestibility of rainbow trout has been verified by Torno et al. [60] and Cleveland et al. [61] also reported it. As a result, they did not observed any positive impact on fish gross energy and lipid content because these compounds played role to modulate proteins responsible for nutrient retention.

## 5 Conclusion

Our results showed that dietary CAR and MEN promoted an increase in growth, feed efficiency and digestibility in Nile tilapia when supplemented either individually or in mixtures; with MIX (1:1) at 200 mg/kg as the optimal supplementation for this species. The plant-based CAR and MEN provided an innovative way to increase feed efficiency and conserving fish meal usage. Moreover, the increased nutrient digestibility provided by dietary CAR and MEN can be considered an environmentally friendly approach to reduce water pollution caused by the less digestible feeds widely used in the current fish farming practices.

## Funding statement

Open access funding provided by Swedish University of Agricultural Sciences.

## Ethical statement

All the protocols have been approved by the “Institutional Review Board Government College University Faisalabad” with Study No: 19666-A, IRB No. 666-A and Ref. No. GCU/ERC/2066-A.

## Data availability

Data will be available on demand.

## CRediT authorship contribution statement

**Aqsa Sharif:** Writing – original draft. **Syed Makhdoom Hussain:** Supervision, Software, Methodology, Investigation, Formal analysis, Data curation, Conceptualization. **Shafaqat Ali:** Writing – review & editing, Investigation, Conceptualization. **Muhammad Rizwan:** Writing – review & editing, Investigation, Conceptualization. **Khalid A. Al-Ghanim:** Writing – review & editing, Investigation, Formal analysis, Data curation. **Jean Wan Hong Yong:** Writing – review & editing, Investigation, Funding acquisition, Data curation.

## Declaration of competing interest

The authors declare that they have no known competing financial interests or personal relationships that could have appeared to influence the work reported in this paper.
